# Cellulolytic and Xylanolytic Microbial Communities Associated With Lignocellulose-Rich Wheat Straw Degradation in Anaerobic Digestion

**DOI:** 10.3389/fmicb.2021.645174

**Published:** 2021-05-25

**Authors:** Mads Borgbjerg Jensen, Nadieh de Jonge, Maja Duus Dolriis, Caroline Kragelund, Christian Holst Fischer, Martin Rosenørn Eskesen, Karoline Noer, Henrik Bjarne Møller, Lars Ditlev Mørck Ottosen, Jeppe Lund Nielsen, Michael Vedel Wegener Kofoed

**Affiliations:** ^1^Department of Biological and Chemical Engineering, Aarhus University, Aarhus, Denmark; ^2^Department of Chemistry and Bioscience, Aalborg University, Aalborg, Denmark; ^3^NIRAS A/S, Aalborg, Denmark; ^4^Danish Technological Institute, Aarhus, Denmark

**Keywords:** anaerobic digestion, hydrolysis, microbial community, microbial adaptation, lignocellulose, fluorometric enzyme assay, biogas, wheat straw

## Abstract

The enzymatic hydrolysis of lignocellulosic polymers is generally considered the rate-limiting step to methane production in anaerobic digestion of lignocellulosic biomass. The present study aimed to investigate how the hydrolytic microbial communities of three different types of anaerobic digesters adapted to lignocellulose-rich wheat straw in continuous stirred tank reactors operated for 134 days. Cellulase and xylanase activities were monitored weekly using fluorescently-labeled model substrates and the enzymatic profiles were correlated with changes in microbial community compositions based on 16S rRNA gene amplicon sequencing to identify key species involved in lignocellulose degradation. The enzymatic activity profiles and microbial community changes revealed reactor-specific adaption of phylogenetically different hydrolytic communities. The enzymatic activities correlated significantly with changes in specific taxonomic groups, including representatives of *Ruminiclostridium*, *Caldicoprobacter*, *Ruminofilibacter*, *Ruminococcaceae*, *Treponema*, and *Clostridia* order MBA03, all of which have been linked to cellulolytic and xylanolytic activity in the literature. By identifying microorganisms with similar development as the cellulase and xylanase activities, the proposed correlation method constitutes a promising approach for deciphering essential cellulolytic and xylanolytic microbial groups for anaerobic digestion of lignocellulosic biomass.

## Introduction

Anaerobic digestion is a well-known biological process for handling and upcycling different waste streams and biomasses to a renewable methane-rich biogas and digestate-fertilizer (Weiland, 2010). The installed global biogas capacity is continuously expanding (Scarlat et al., 2018), and biomethane is expected to be an essential energy source in the fossil-free energy system (Wall et al., 2018). Feedstocks for biogas production are mainly based on low-cost organic waste streams like manure, sludge, and organic household waste. Lignocellulosic residues from the agricultural sector, such as deep litter and straw residues, constitute a highly abundant type of biomass whose use in anaerobic digestion is essential to realize the technology’s full societal potential for renewable methane production (Sawatdeenarunat et al., 2015). However, the recalcitrant structure of lignocellulose protects the cellulosic and hemicellulosic polymers from microbial degradation and constitutes an economic and technical barrier to anaerobic digestion of lignocellulosic biomasses. Anaerobic digestion of lignocellulosic biomasses consequently relates to low methane production rates (MPRs), entailing long solid retention times or excessive reactor volume to obtain high methane yields (Achinas et al., 2017). Microbially secreted hydrolases catalyze the initial hydrolysis of cellulosic and hemicellulosic polymers, which is considered the rate-limiting step in anaerobic digestion of recalcitrant lignocellulosic substrates (Noike et al., 1985; Sambusiti et al., 2014). Most research has consequently focused on developing pretreatment strategies that accelerate the biological degradation of lignocellulose by breaking up the recalcitrant structure and exposing the cellulosic and hemicellulosic polymers to enzymatic attack (Ahring et al., 2015; Feng et al., 2017). The MPRs and methane yields from cellulosic and lignocellulosic biomass depend on the origin of the inoculum, as the specific microbial community, activity, and ability to adapt to lignocellulosic biomass degradation is shaped by the operating conditions applied in the anaerobic digester (Sun et al., 2016; Koch et al., 2017; Liu et al., 2017). Obtaining a more thorough understanding of the involved microbial and functional dynamics during anaerobic digestion of lignocellulosic biomass can potentially promote methane production directly in the anaerobic digester and reduce the need for costly pretreatment technologies accordingly (Hu et al., 2011).

High-throughput next-generation sequencing techniques such as 16S rRNA gene amplicon sequencing have provided tools to characterize the microbial communities in anaerobic digesters in terms of phylogeny and potential functions, enabling monitoring of changes in the microbial communities in response to different operational conditions (de Jonge et al., 2017, 2020). These properties have fueled the idea of developing microbial control systems and optimizing the conventional anaerobic digestion process based on the identification of microbial process indicators (Carballa et al., 2015; De Vrieze, 2020). The development of microbial-based control strategies is still troubled by the significant shortage of fundamental knowledge on the active microbial community and microbial indicators, which are influenced by a range of process conditions (De Vrieze et al., 2018). Firstly, a large part of the microbiome in anaerobic digesters is still unknown and therefore only classified on higher taxonomic ranks (Campanaro et al., 2016). Secondly, potential functions indicated by metagenomic analyses do not necessarily translate into important operational parameters describing substrate turnover (Hassa et al., 2018). Different approaches are being developed to decipher the active microbiome, e.g., using multi-omics approaches (Mosbæk et al., 2016; De Vrieze et al., 2018), or microbial fingerprinting techniques to map the presence or expression of specific genes (Zhang et al., 2014; Müller et al., 2016). Combining bulk activity tests, such as organic degradation and methane production rates, with microbial community data has also been shown as a promising approach for identifying microbial groups important to the degradation of specific substrates (Regueiro et al., 2012). Cellulolytic bacteria have been identified at different phylogenetic levels by correlating degradation rates of lignocellulosic straw and cellulose with changes in specific genes encoding hydrolases involved in cellulose degradation (Sun et al., 2016). This study uses a new approach to identify potential key microbial communities involved in lignocellulose hydrolysis in anaerobic digestion by correlating changes in microbial community structures directly to changes in cellulase and xylanase activities. Cellulases and xylanases catalyze the hydrolysis of cellulose and xylan (hemicellulose), respectively, which are abundant lignocellulosic polymers. The hydrolytic bacterial community expressing these enzymes is consequently of particular importance to the MPR during anaerobic digestion of lignocellulosic material.

The objective of the present study was to investigate (i) how different types of digestate inocula respond and adapt to increased feeding with lignocellulose-rich wheat straw, based on changes in microbial community structures, activities of cellulase and xylanase, and the MPR; and from these data (ii) identify potential hydrolytic microorganisms by correlating the enzymatic activities and the microbial community compositions. Mixtures of wheat straw and inoculum-specific biomass material were used as model substrate and fed to lab-scale CSTRs inoculated with digestate from different manure- and sludge-based full-scale digesters. The hydrolytic adaptation was characterized for three different inoculum types to demonstrate the broad applicability of the proposed correlation method.

## Materials and Methods

### Inoculum Characterization

Digestates from three different full-scale digesters were used as inocula in lab-scale reactors within few hours after their collection at the plants. All biomasses were sieved through a 1 mm mesh prior to inoculation and subject to an acclimation period of 11 days before the period of straw-feeding (section “Reactor Operation”). The inocula were expected to have different capabilities for degrading lignocellulosic substrates, reflected by the differences in substrate types and operational conditions of the full-scale digesters (Table 1). R1 (Aarhus University Foulum, Denmark) was expected to be highly adapted to digest lignocellulosic biomass since it was operated at thermophilic conditions and its primary substrates included cattle, and pig manure as well as straw and deep litter. R2 (Maabjerg Energy Center, Denmark) treated a larger mix of biomasses, including industrial waste, cattle, and pig manure, and deep litter. R3’s (Billund Vand and Energi, Denmark) primary substrates were pre-treated sludge and industrial waste. R2 and R3 were both operated at mesophilic temperatures.

**TABLE 1:** Operational information of the sampled full-scale digesters and the physiochemical characteristics of the biomass samples used as inoculum in the lab-scale reactors.

**Operational parameters**	**R1**	**R2**	**R3**
Hydraulic retention time (d)	13	26	30
Organic loading rate (kg VS ⋅ m^–3^ ⋅ d^–1^)	8	2.5	n.a.
Reactor volume (m^3^)	1,200	3,750	2,800
Methane content (%)	58	61	69
Methane production rate (m^3^ ⋅ m^–3^ ⋅ d^–1^)	2.2	0.8	1.6
Temperature (°C)	52	37	37
Substrates	Cattle manure Pig manure, Deep litter Straw Grasses	Industrial waste Cattle manure Pig manure Deep litter Straw	Sewage sludge Municipal waste Industrial waste
Inoculum	R1	R2	R3
TS (%)	3.9 ± 0.0	3.3 ± 0.2	3.6 ± 0.0
VS (%)	2.8 ± 0.0	2.2 ± 0.1	2.4 ± 0.0
VFA (mg ⋅ L^–1^)	248 ± 14	677 ± 96	727 ± 9
NH_4_^+^ (g ⋅ L^–1^)	1.5 ± 0.01	3.9 ± 0.4	2.0 ± 0.01
COD (g ⋅ L^–1^)	45.2 ± 11.4	35.6 ± 8.8	25.8 ± 4.6
pH	7.5 ± 0.1	7.6 ± 0.1	7.4 ± 0.0

*n.a. = not available.*

### Reactor Operation

Six continuous stirred tank reactors (CSTR) (1.8 L working volume, 2 L total volume) (Bioprocess Control AB, Sweden) were operated anaerobically for 134 days. The lab-scale reactors were operated in duplicates and inoculated with digestates from either R1, R2, or R3 using temperature conditions similar to the full-scale systems (Table 1). The feeding period started on day 11 and ended on day 81. The hydraulic retention time (HRT) was 20 days, and the reactors were fed three times per week using mixtures of degassed reactor-specific slurry material and wheat straw. The wheat straw was down-sized (<10 mm) using a hammer mill. Its fiber composition was determined previously as a percentage of dry weight: hemicellulose 30.4 ± 0.8 w%, cellulose 44.8 ± 0.8 w%, and lignin 7.2 ± 0.5 w%. The organic loading rate (OLR) was defined solely based on the wheat straw and initially set to 2.0 g volatile solids (VS) ⋅ L^–1^ ⋅ day^–1^, but increased to 3.0 g ⋅ VS ⋅ L^–1^ ⋅ day^–1^ from day 32, i.e., after one HRT. Stirring and temperature remained constant for another 64 days after the feeding period had ended to study the influence of starvation on enzymatic activities. Methane production was monitored continuously using the AMPTS II (Bioprocess Control AB, Sweden). MPR is presented with units of L ⋅ L^–1^ ⋅ day^–1^ and shown as a weekly average to even out fluctuations in gas production caused by the discontinuous feeding three times per week. Samples for physicochemical and microbial community characterizations were withdrawn at weekly intervals and stored at −20°C until further analysis.

### Physicochemical Reactor Characterizations

Total solids (TS) and volatile solids (VS) were determined according to standard procedures (Rice et al., 2017). Chemical oxygen demand (COD) and ammonium concentrations (NH_4_^+^) were determined using commercial kits according to the manufacturer’s protocol (HACH^®^, Germany). Volatile fatty acid (VFA) concentrations were determined using gas chromatography equipped with a HP-INNOWAX column (30 m, 0.25 mm, 0.25 μm) (Agilent Technologies) and a flame ionization detector using helium as carrier gas.

### Cellulase and Xylanase Activities

The activities of extracellular cellulases and xylanases were consistently determined in one of the duplicate reactors for R1, R2, and R3. The enzymatic activities were determined every week during the period with organic feeding (day 11–81) and three times in the subsequent starvation period (day 82–145). Enzyme activities were determined using a fluorometric assay, where slurry samples from the individual reactors were incubated with 4-methylumbelliferone (MUF)-linked cellobioside and xylopyranoside compounds (Sigma-Aldrich, Denmark). Specifically, 4-methylumbelliferyl β-D-cellobioside (MUF-cellobioside), and 4-methylumbelliferyl-β-D-xylopyranoside (MUF-xylopyranoside) were used as fluorometric substrates that resemble breakdown products of polymeric cellulose and xylan, respectively. MUF-labeled substrate analogs have previously been used to analyze hydrolase activities in different environmental systems, including wetlands (Freeman et al., 1995), soil (Darrah and Harris, 1986), and sediments (King, 1986), and was here adopted for the biogas process. Preliminary incubations with commercial enzymes showed that MUF-cellobioside was hydrolyzed mainly by endoglucanases and to a minor extent by β-glucosidase. MUF-xylopyranoside was hydrolyzed mainly by β-xylosidase and to a minor extent by endoglucanase (Data not shown).

Slurry was withdrawn from the laboratory biogas reactor and prepared for enzymatic analyses by homogenizing the sample with a high-speed dispersing instrument (T 10 basic Ultra Turrax; IKA). The remaining non-pipettable particulates were removed by centrifugation at 300 × g for 60 s and filtering (800 μm mesh). Before the addition of the MUF-linked substrates, the homogenized slurry was diluted in 50 mM Tris-HCl (pH 7.5) to a final concentration of 0.5 or 1% for (i) analyzing conversion of the MUF-linked substrates at non-substrate limiting conditions (V_*max*_); while (ii) avoiding MUF concentrations exceeding the linear range of the MUF standard curve (due to high rates of hydrolysis). The two MUF-labeled model substrates were added to individual slurry samples in two concentrations to assess if the hydrolysis rates were independent of substrate concentrations. The final concentration was 0.28 and 0.14 mmol ⋅ L^–1^ for MUF-cellobioside, and 0.11 and 0.06 mmol ⋅ L^–1^ for MUF-xylopyranoside. Negative controls were prepared by heat-inactivating the slurry at 100°C for 10 min prior to the homogenization step described above. The water lost due to evaporation was added to the slurry after boiling. MUF standards were prepared in both active and heat-inactivated slurry samples. These standards were analyzed along with both active samples and the negative controls.

All samples were transferred to a black flat-bottomed 96-well microplate (Greiner Bio-One, Germany) and analyzed in duplicates at reactor-specific temperatures using a microplate reader system (Varioskan LUX, Thermo Fischer Scientific Inc., United States). Excitation and emission wavelengths of MUF were 355 and 445 nm, respectively, and fluorescence from released MUF was measured every fifth minute for at least 2 h. Hydrolase activities are reported as the initial linear hydrolysis rate (zero-order kinetics), averaged for the two substrate concentrations.

It is important to note that the enzymatic activity in R1 was assayed at 45°C, despite reactor operation at 52°C. This discrepancy was due to a temperature constraint of the plate reader incubator. The actual enzymatic activities of R1 were, therefore, likely higher than measured here due to kinetic effects. Samples from R2 and R3 were analyzed at 37°C, similar to reactor conditions.

### DNA Extraction and 16S rRNA Gene Amplicon Sequencing

Slurry samples were withdrawn for DNA sequencing on a weekly basis throughout the feeding period. Genomic DNA was extracted from 300 μL of digester slurry using the FastDNA^TM^ SPIN Kit for Soil (MP Biomedicals, Inc.) according to the manufacturer’s recommendation with minor alterations; bead-beating was performed in 4 cycles of 40 s at 6 m ⋅ s^–1^, and elution was performed in 60 μL DES buffer. DNA quantity and quality were assessed using a Qubit 2.0 fluorometer with Qubit dsDNA BR Assay kit (Thermo Fisher Scientific, United States) and TapeStation 2200 with Genomic DNA ScreenTapes (Agilent, United States). The V4 region of the 16S rRNA gene was amplified as previously described (Albertsen et al., 2015). Obtained libraries were purified using the AMPure XP bead protocol with a sample:bead ratio of 0.8 (Beckmann Coulter, United States). Library quantity and quality were determined using Quant-iT dsDNA HS Assay Kit (Thermo Fischer Scientific, United States) and D1000 ScreenTapes (Agilent, United States). The libraries were sequenced on a MiSeq (Illumina, United States) in equimolar concentrations using reagent kit v2 (1 × 300). The raw sequencing data was processed into amplicon sequencing variants (ASVs) using the AmpProc pipeline (version 5.1)^1^ in single read mode. Taxonomy was assigned using MiDAS (v4.6) as the reference database (McIlroy et al., 2017).

### Data Analysis

R version 3.6.1 (R Core Team, 2016) and RStudio version 1.2.5001^2^ were used for all statistical and microbial community analyses. Alpha diversity was measured using the Chao1 (Chao, 1984) and Shannon-Weaver (Shannon, 1948) indices, and beta diversity was visualized using correspondence analysis (CA). Microbial compositions were visualized using heatmaps. The overall link between the microbial community development and the enzymatic activities was investigated using Redundancy Analysis (RDA). ASVs with a strong link to the enzymatic activities were extracted from the dataset as follows: All ASVs were subjected to the Kolmogorov-Smirnov test (Massey, 1951) to select ASVs with a distribution similar to that of the measured enzymatic activities. The ASVs with the strongest relationship to the enzymatic rates were selected as hydrolytic candidates based on a subsequent linear regression analysis of the relative abundances of the selected ASVs, normalized by the enzymatic activity. Pearson’s correlations were used to correlate operational parameters. All other visualizations were generated using ggplot2 (Wickham, 2016).

## Results

### Performance of Laboratory Reactors

**FIGURE 1:**
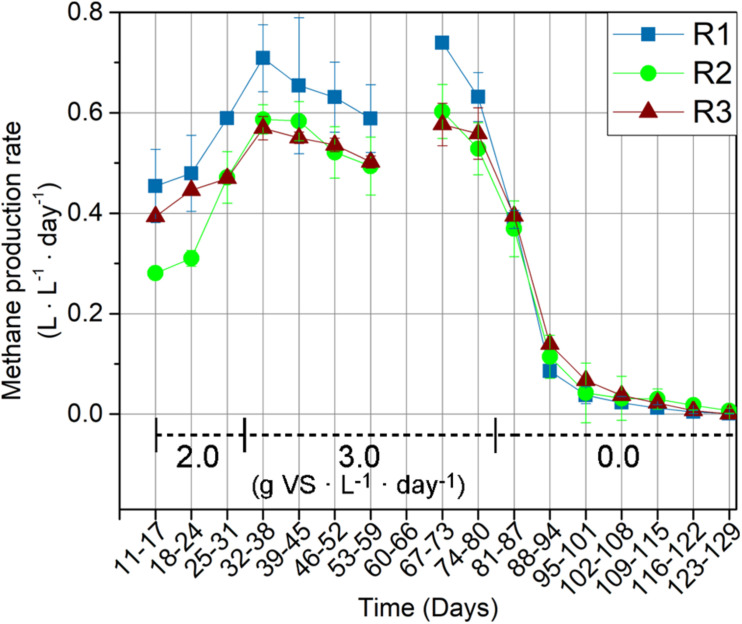
Methane production rates for the thermophilic reactor (R1), and mesophilic reactors (R2 and R3). MPRs are presented as weekly average. The reactors were fed with wheat straw from day 11–81 and organic loading rates (g VS ⋅ L^–1^ ⋅ day^–1^) are indicated on the graph. The MPR was not recorded during days 60–66 due to a technical error.

The thermophilic R1 exhibited the highest MPR compared to the mesophilic R2 and R3 throughout the feeding period (day 11–81) (Figure 1). The MPR in R1 was 0.45 ± 0.07 L ⋅ L^–1^ ⋅ d^–1^ during the first week of operation (day 11–17), corresponding to a 62 and 15% higher MPR compared to those in R2 and R3, respectively. The MPR in R1 was 20% (R2) and 13% (R3) higher in the last week of the feeding period (day 74–80), where it amounted to 0.63 ± 0.05 L ⋅ L^–1^ ⋅ d^–1^ in R1 compared to 0.53 ± 0.05 L ⋅ L^–1^ ⋅ d^–1^ and 0.56 ± 0.05 L ⋅ L^–1^ ⋅ d^–1^ in R2 and R3, respectively. Among the mesophilic reactors, R2 and R3, the former exhibited a lower MPR during the first 14 days of operation (day 11–24). However, R2 and R3 exhibited similar MPRs from the third week of operation (day 25–31) and throughout the remaining experimental period.

An initial lag phase was observed in all reactors, evidenced by the consistent increases in MPRs during the first 1.5 HRT (day 11–38) (Figure 1). The MPR increased by 56, 109, and 45% in R1, R2, and R3, respectively, between days 11–18 (week 1 of feeding) and days 32–38 (week 4). The OLR was 2.0 g VS ⋅ L^–1^ ⋅ day^–1^ up to and including day 31 (i.e., the first HRT) and increased to 3.0 g VS ⋅ L^–1^ ⋅ day^–1^ hereafter to push adaptation of the hydrolytic community further. The increase in OLR was accompanied by increases in the MPRs only in the first week (days 32–38), followed by a decreasing trend for all reactors in the following weeks. However, the decrease in MPR was not permanent. The MPRs thus increased towards the end of the feeding period, which ended at day 81. MPRs during days 60–66 were not recorded due to technical issues. Overall, the 50% increase in OLR at day 32 resulted in MPR increases of 7, 12, and 19% in R1, R2, and R3, respectively, when comparing the final week of feeding (days 74–80) with the period before the OLR increase (days 25–31). The MPR decreased significantly in all reactors after feeding was stopped at day 81 (Figure 1).

The stable performance of R1 was also indicated from process indicators in the digestate, where pH, VFAs, and the COD remained relatively stable (Table 2). pH values were 7.3 ± 0.2 in R1 and R2 and 7.1 ± 0.2 in R3 throughout the experiment. In R2, the reactor subjected to the most significant increase in MPR, VFA levels increased throughout the experimental period, from 656 ± 94 mg ⋅ L^–1^ at day 18 to 2,563 ± 812 mg ⋅ L^–1^ at the last day of feeding (day 81). The COD in R2 increased from 28.9 ± 6.2 g ⋅ L^–1^ at day 18 to 39.2 ± 4.9 g ⋅ L^–1^ at day 81. In R3, there was a temporary increase in VFA levels around day 62. The COD in R3 increased from 25.3 ± 2.5 g ⋅ L^–1^ on day 18 to 35.6 ± 5.6 g ⋅ L^–1^ on day 81, similar to the final COD in R1 (Table 2).

**TABLE 2:** Physicochemical parameters of effluent samples from the duplicate lab-scale reactors.

**R1**					
*Day*	*18*	*41*	*62*	*81*	*141*

pH	7.4 ± 0.0	7.2 ± 0.0	7.2 ± 0.0	7.1 ± 0.1	8.2 ± 0.0
VFA (mg ⋅ L^–1^)	310 ± 70	273 ± 41	324 ± 12	450 ± 60	−
NH_4_^+^ (g ⋅ L^–1^)	1.2 ± 0.04	0.6 ± 0.05	0.4 ± 0.01	0.4 ± 0.01	−
COD (g ⋅ L^–1^)	44.9 ± 4.2	35.1 ± 5.1	34.8 ± 4.8	35.7 ± 3.3	−

**R2**					
pH	7.5 ± 0.0	7.3 ± 0.1	7.3 ± 0.1	7.2 ± 0.0	7.9 ± 0.0
VFA (mg ⋅ L^–1^)	656 ± 94	1,232 ± 505	1,345 ± 90	2,563 ± 812	−
NH_4_^+^ (g ⋅ L^–1^)	2.7 ± 0.0	1.4 ± 0.05	1.1 ± 0.2	1.2 ± 0.1	−
COD (g ⋅ L^–1^)	28.9 ± 6.2	31.0 ± 0.9	30.1 ± 2.4	39.2 ± 4.9	−

**R3**					
pH	7.3 ± 0.1	7.0 ± 0.0	7.0 ± 0.0	6.9 ± 0.0	7.9 ± 0.0
VFA (mg ⋅ L^–1^)	649 ± 127	680 ± 18	2,138 ± 1,106	668 ± 43	−
NH_4_^+^ (g ⋅ L^–1^)	1.5 ± 0.03	0.8 ± 0.04	0.6 ± 0.0	0.4 ± 0.0	−
COD (g ⋅ L^–1^)	25.3 ± 2.5	34.9 ± 4.4	32.6 ± 0.0	35.6 ± 5.6	−

*R1 is thermophilic and R2 and R3 are mesophilic reactors.*

### Cellulase and Xylanase Activities in Laboratory Reactors

**FIGURE 2:**
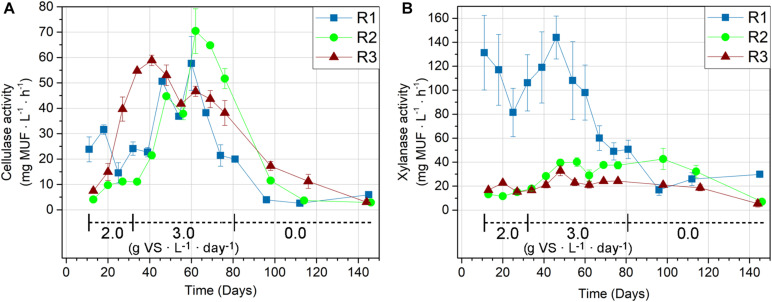
Cellulase **(A)** and xylanase **(B)** activities given by MUF release rate in the thermophilic R1, and the mesophilic reactors R2 and R3. The reactors were fed with wheat straw from day 11–81 and organic loading rates (g VS ⋅ L^–1^ ⋅ day^–1^) are indicated on the graphs.

The cellulase (Figure 2A) and xylanase (Figure 2B) activities were initially highest in the thermophilic R1, supporting its higher MPR compared to the mesophilic reactors, R2 and R3 (Figure 1). The initial enzymatic activities in R1 were thus 5.9 (cellulase) and 10.0 (xylanase) times higher compared to the activities in R2, and 3.2 (cellulase) and 7.9 (xylanase) times higher than the activities in R3. The enzymatic activities in R1 were likely even higher than measured since the enzymatic analysis was technically constrained to 45°C, whereas R1’s operating temperature was 52°C. Furthermore, the xylanase activity measurements in R1 were not determined at V_*max*_ conditions, explaining the higher standard deviations for these measurements. The actual differences between the xylanase activities in R1 and the two mesophilic reactors are hence likely to be larger than measured here (Figure 2B).

*Cellulase*. The largest increases in cellulase activity were observed in R2 and R3. The cellulase activity in R3 increased immediately after the experimental start and continued until day 41 (corresponding with 1.5 HRT), where it was 7.9-fold higher compared to the initial measurement (Figure 2A). The cellulase activity in R2 showed a slower response compared to R3. Compared to R3, the R2 cellulase activity increase was modest during the first hydraulic retention time with straw feedings (days 11–34). However, the increase in OLR from 2.0 to 3.0 g VS ⋅ L^–1^ ⋅ day^–1^ at day 32 coincided with a significant increase in R2 cellulase activity in the following period. The R2 cellulase activity peaked at day 62 (corresponding to 2.5 HRT) with 70.4 ± 8.8 mg MUF ⋅ L^–1^ ⋅ h^–1^, equivalent to a 17.4-fold increase compared to the initial activity. Despite the consistent increase in MPR by 56% (Figure 1), the R1 cellulase activity remained relatively stable during the first 28 days of straw feeding (day 11–39). However, the activities in R1 increased to 1.5–2.4 times the initial cellulase activity in the subsequent period (day 46–67) before declining to 84–90% of the original activity in the last week of feeding (days 74 and 81). R2 and R3 were subject to similar decreases in cellulase activity within the feeding period, but the onset varied. R3 cellulase activity started to decline after day 41 (corresponding to HRT 1.5), while the decrease in activity in R1 and R2 started after day 60 and 62 (corresponding to HRT 2.5), respectively.

*Xylanase*. The xylanase activities in R1 remained within 62–110% of the initial activity during the first 35 days (days 11–46) of straw feeding (Figure 2B). The R1 xylanase activity was 8–10-fold higher than in R2 and R3 for the first measurement. However, the R1 xylanase activity started declining after day 46, ending at 37% of its initial activity on the last day of feeding (day 81). For the mesophilic reactors, the xylanase activity increased more pronouncedly in R2 compared to R3, stabilizing at ∼3-fold and 1.5-fold gains in the feeding period, respectively, compared to the initial measurements. The xylanase activities in R2 and R3 did not decrease until the starvation period, which began on day 81.

*Response to starvation*. The cellulase and xylanase activities generally declined after the feeding period ended (day 81 and onward), but the development was both enzyme- and reactor-dependent. The cellulase activities continued to decrease in all reactors after the feeding period ended. Still, the cellulases retained 30% (R1), 6% (R2), and 8% (R3) of their activities measured prior to starvation after more than 60 days without feeding (Figure 2A). The decline in xylanase activities was slower compared to the decline in cellulase activities in all reactors. The retained xylanase activity was 59% (R1), 19% (R2), and 22% (R3) after more than 60 days without feeding (Figure 2B).

*Correlation with MPR*. The Pearson’s correlation test was used to indicate if the cellulase and xylanase activities were limiting the MPRs during the feeding period. The MPRs had a stronger correlation with cellulase activities (*r* = 0.66–0.80) compared to xylanase activities (*r* = 0.27–0.67) in all reactors, indicating the importance of cellulases for the MPR. Focusing on the first 1.5 HRT of straw feeding (day 11–41; five data points), where the MPRs generally increased: the cellulase and xylanase activities showed weak, negative correlation with MPR in R1 (*r* = −0.34 for cellulase, *r* = −0.42 for xylanase), compared to strong positive correlations of both cellulase and xylanase in R2 (*r* = 0.76 for cellulase, *r* = 0.77 for xylanase) and cellulase in R3 (*r* = 0.95 for cellulase and *r* = 0.05 for xylanase). The MPR in R1 was thus not indicated to be limited by the activity of the measured hydrolases. In contrast, the MPR in R3 was strongly associated with the cellulase activity during the initial period. The cellulase activities were conversely highly correlated with MPRs in all reactors from day 46 and onward (eight data points for R2 and R3; nine data points for R1), with correlation coefficients of 0.89 (R1), 0.98 (R2), and 0.96 (R3).

### Microbial Community Compositions During Feeding

Amplicon sequencing of the 16S rRNA gene yielded high-quality reads for 60 of the 70 sequenced samples. Samples generating less than 10,000 reads were removed from analysis based on a rarefaction curve (data not shown). A total of 3,269,716 reads were generated across 60 samples and 9,273 ASVs, with an average of 54,495 ± 21,312 reads per sample. Community richness (Chao1 index) was measured at an average of 4,133 ± 456 ASVs per sample and was stable across 82 days of monitoring in the sampled duplicate reactors (data not shown). The highest richness was observed in the mesophilic R3 with an average of 4,503 ± 306 ASVs per sample. The lowest richness was observed in the thermophilic manure-based R1, with an average of 3,722 ± 234 ASVs per sample. Evenness (Shannon-Weaver index) in the reactors was measured at 6.51 ± 0.29 across 82 days of sampling and was highly similar between the three reactor types (6.49–6.53).

Correspondence Analysis (CA) was used to examine the development in microbial community composition in the three reactors during the feeding period (Supplementary Figure 1). Three distinct clusters representing the different duplicate reactors formed, indicating that distinct microbial communities developed from each inoculum. Tight clusters were formed for R1 and R3, while a larger development along the secondary axis was observed for R2. The specific microbial community compositions in the three duplicate reactors were explored using heatmaps (Supplementary Figures 2–4):

*R1*. The relative abundance of several microbial populations increased from the start toward the end of the feeding period, including *Cloacimonadaceae* group W5 (3.5–18.1% of total reads), “*Candidatus* Caldatribacterium” (1.4–7.5% of total reads), *Methanobacterium* (0.2–6.7% of total reads), and *Methanosarcina* (0.5–4.8% of total reads). Other microbial groups decreased in abundance, including *Defluviitoga* (24.2–0.4% of total reads), *Clostridia* order MBA03 (from 29.1 to 4.5% of total reads), and several uncharacterized microorganisms (Supplementary Figure 2).

*R2*. The development of the microbial community in R2 was characterized by an increase in the relative abundance of *Rikenellaceae* group DMER64 (1.0–24.1% of total reads), two representatives of *Dysgonomonadaceae* (0.7–9.8% of total reads), *Trichococcus* (0–3.8% of total reads), and *Ruminiclostridium* (0.2–3.7% of total reads). The relative abundance of *Bacteroidetes* representatives ST-12K33 and *Bacteroidales* UCG-001 had decreased by the end of the feeding period (49.9–13.9% of total reads). However, the relative abundance of *Bacteroidales* UCG-001 increased initially and peaked at 19.7% by day 42. *Bacteroidales* UCG-001 thus showed a similar developmental pattern as *Enterococcus*, whose relative abundance increased throughout the first 56 days (from 0.9 to 21.1%) before reaching 3.4% by the end of the feeding period. The most abundant methanogenic populations belonged to *Methanothrix* and *Methanosarcina*, whose relative abundance, respectively, increased (0.5–1.1%) and decreased (1.3–0.2%) throughout the feeding period (Supplementary Figure 3).

*R3*. The relative abundance of the genus *Trichococcus* increased from < 0.1 to 26.1% of total reads at day 48 but decreased to 9.9% of total reads at the end of the feeding period. Other taxonomic groups showing an increase in relative abundance were *Bacteroidales* group UCG-001 (5.1–18.7% of total reads), “*Candidatus* Cloacimonas” (1.9–5.2% of total reads), *Ruminiclostridium* (0.9–6.2% of total reads at day 78), and *Roseimarinus* (<0.1–1.4% of total reads). In contrast, a decrease was observed in the relative abundance of *Cloacimonadaceae* group W5 (31.7–3.1% of total reads), *Bacteroidetes* group vadinHA17 (4.4–1% of total reads), *Methanothrix* (5.3–0.7% of total reads), and *Sedimentibacter* (3–0.6% of total reads) (Supplementary Figure 4).

### Linking Community Composition to Cellulase and Xylanase Activities

**FIGURE 3:**
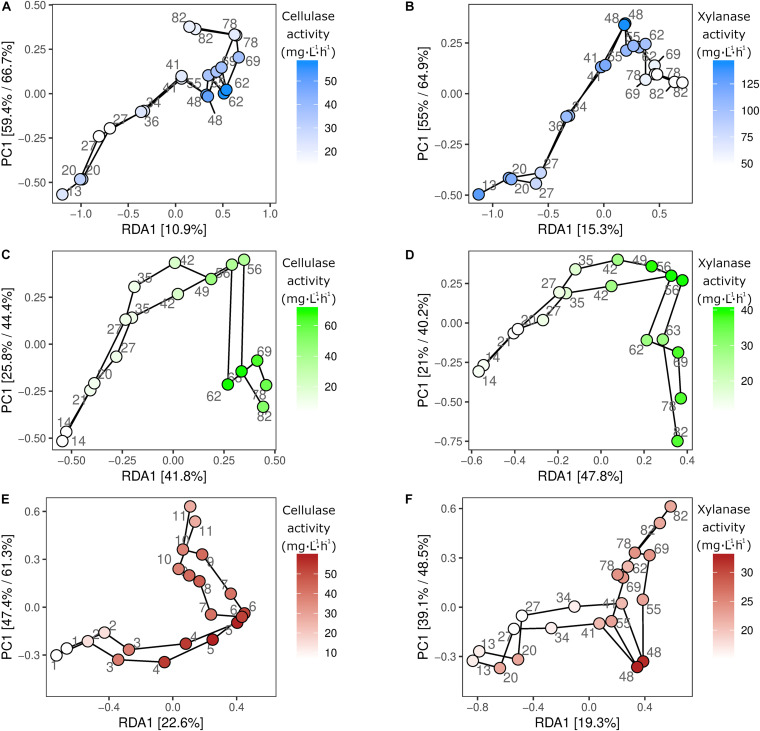
Redundancy analysis. RDA analysis of the sampled duplicate reactors R1 **(A,B)**, R2 **(C,D)**, and R3 **(E,F)**, constrained by either cellulase **(A,C,E)** or xylanase **(B,D,F)** activity. Samples are colored by enzymatic activity and a line is drawn between consecutive sampling points in the time series. The individual ASVs are shown on the models as gray dots. R1 is thermophilic, and R2 and R3 are mesophilic reactors.

The microbial community composition changes were examined in relation to the enzymatic profiles to identify individual subpopulations with potential hydrolytic functions. The potential link between microbial identity and hydrolytic function was established by correlating the changes in relative microbial abundance to those of the enzymatic activities (Figure 3). The correlation between enzymatic and microbial community profiles was significant in all duplicate reactors for both cellulase and xylanase. The strongest relationship between microbial community compositions and enzymatic activities was observed in R2 for both cellulase (*p* = 0.001, *R*^2^ = 0.82), and xylanase (*p* = 0.001, *R*^2^ = 0.89) (Figures 3C,D). A significant, but weaker relationship between the microbial community and the cellulase (*p* = 0.006, *R*^2^ = 0.44) and xylanase (*p* = 0.009, *R*^2^ = 0.46) activities was observed in R1 (Figures 3A,B). In R3, the cellulase activity (*p* = 0.001, *R*^2^ = 0.71) had a stronger relationship to the microbial community compared to xylanase activity (*p* = 0.008, *R*^2^ = 0.41) (Figures 3E,F).

To extract ASVs with a similar development as the enzymatic activities and thereby identify taxonomic groups with potential hydrolytic function, the Kolmogorov-Smirnov test was applied to z-score normalized abundance and activity values. The largest number of ASVs of interest was observed in R2, where 258 and 293 ASVs showed similar development to the cellulase and xylanase activity, respectively. The number of identified ASVs ranged 61–152 ASVs in R1 and R3 (data not shown). The 25 ASVs with the strongest correlation to the cellulolytic and xylanolytic activities were selected from each reactor and enzyme combination for further examination (Tables 3–5). These candidates were of low abundance within the microbial community, having a relative abundance of 0.01–0.5% of total reads per sample during the experimental period of 82 days (Supplementary Figure 5). In all reactor and enzyme combinations, the collective abundance of the candidate ASVs increased over time, except for R2 and cellulase (decrease from 1.14% at day 13 to 0.43% at day 78). The greatest increase in relative abundance was seen for R3 and cellulase, where the candidate ASVs made up 0.18 and 1.25% of total reads at day 13 and 78, respectively.

**TABLE 3:** The 25 ASVs with behavior most similar to the enzymatic activities in the thermophilic R1.

**R1—Cellulase**		**R1—Xylanase**	
Zotu102	Ruminofilibacter	Zotu1334	Lentimicrobiaceae
Zotu1108	Lentimicrobiaceae	Zotu1665	Lentimicrobiaceae
Zotu1109	Methanoculleus thermophilus	Zotu1682	Romboutsia
Zotu1272	Lentimicrobiaceae	Zotu1892	Clostridiales
Zotu1300	Lentimicrobiaceae	Zotu2034	Caldicoprobacter
Zotu1334	Lentimicrobiaceae	Zotu2056	Ruminococcaceae
Zotu1617	Firmicutes	Zotu2076	Lentimicrobiaceae
Zotu1666	Clostridiales Family XIII	Zotu2083	Turicibacter sanguinis
Zotu175	Ca. Caldatribacterium	Zotu2195	Dethiobacter
Zotu1780	Ruminococcaceae	Zotu2287	Ruminococcaceae
Zotu1829	Bacteria	Zotu2358	Turicibacter sanguinis
Zotu1911	Ruminiclostridium	Zotu2472	Ruminococcaceae UCG-012
Zotu2065	Caldicoprobacter	Zotu2921	Clostridia order D8A-2
Zotu2128	Clostridia	Zotu2927	Ruminococcaceae UCG-012
Zotu2276	Ruminococcaceae UCG-010	Zotu3042	Clostridia order D8A-2
Zotu2313	Tepidanaerobacter	Zotu3158	Caldicoprobacter
Zotu2325	Tepidanaerobacter	Zotu3185	Caldicoprobacter
Zotu2369	Sedimentibacter	Zotu3296	Clostridiales
Zotu2400	Pseudobacteroides	Zotu378	Lentimicrobiaceae
Zotu465	Proteiniphilum	Zotu5675	Clostridia order MBA03
Zotu4805	Clostridia order MBA03	Zotu6753	Clostridiales Family XI
Zotu510	Proteiniphilum	Zotu6757	Clostridia order MBA03
Zotu5675	Clostridia order MBA03	Zotu6994	Clostridia order MBA03
Zotu6503	Clostridia order MBA03	Zotu8202	Clostridia order MBA03
Zotu802	Ruminiclostridium	Zotu9440	Ruminiclostridium

*The best possible taxonomic classification is given for each ASV.*

**TABLE 4:** The 25 ASVs with behavior most similar to the enzymatic activities in the mesophilic R2.

**R2—Cellulase**		**R2—Xylanase**	
Zotu1076	Pedosphaeraceae	Zotu1019	Dysgonomonadaceae
Zotu1199	Pedosphaeraceae	Zotu1190	Pedosphaeraceae
Zotu1241	Pedosphaeraceae	Zotu1312	Pedosphaeraceae
Zotu1282	Spirochaetaceae	Zotu1430	Spirochaetaceae
Zotu1312	Pedosphaeraceae	Zotu1510	Spirochaetaceae
Zotu1559	Ruminiclostridium	Zotu1647	Spirochaetaceae
Zotu1647	Spirochaetaceae	Zotu1652	Ruminiclostridium
Zotu1734	Dysgonomonadaceae	Zotu2071	Acholeplasma
Zotu2094	Dysgonomonadaceae	Zotu2665	Ruminiclostridium
Zotu2330	Ruminococcaceae	Zotu2800	Ruminococcaceae UCG-010
Zotu2591	Bacteroidales	Zotu2852	Ruminococcaceae
Zotu2648	Ruminiclostridium	Zotu2888	Christensenellaceae R-7 group
Zotu2665	Ruminiclostridium	Zotu2896	Ruminococcaceae UCG-010
Zotu2784	Ruminococcaceae	Zotu3770	Acholeplasma
Zotu2800	Ruminococcaceae UCG-010	Zotu3777	Lachnospiraceae
Zotu3428	Ruminococcaceae	Zotu4154	Christensenellaceae R-7 group
Zotu352	Sedimentibacter	Zotu4162	Acholeplasma
Zotu3654	Ruminococcaceae UCG-010	Zotu477	Sedimentibacter
Zotu3777	Lachnospiraceae	Zotu5865	Synergistaceae
Zotu4054	Ruminococcaceae	Zotu6002	Bacteroidales
Zotu4162	Acholeplasma	Zotu7170	Clostridiales Family XI
Zotu4233	Lachnospiraceae	Zotu7932	Christensenellaceae
Zotu7170	Clostridiales Family XI	Zotu8126	Peptococcaceae
Zotu8216	Clostridiales Family XI	Zotu8367	Synergistaceae
Zotu8367	Synergistaceae	Zotu9261	Thermovirga

*The best possible taxonomic classification is given for each ASV.*

**TABLE 5:** The 25 ASVs with behavior most similar to the enzymatic activities in the mesophilic R3.

**R3—Cellulase**		**R3—Xylanase**	
Zotu1031	Acidobacteria	Zotu1094	Petrimonas mucosa
Zotu1071	Clostridia order MBA03	Zotu1175	Petrimonas mucosa
Zotu1138	Ca. Caldatribacterium	Zotu1215	Treponema 2
Zotu1277	Treponema 2	Zotu1245	Mesotoga infera
Zotu1291	Acidobacteria	Zotu1262	Ca. Caldatribacterium
Zotu1499	Acidobacteria	Zotu1292	Treponema 2
Zotu1555	Bacteroidales group vadinHA17	Zotu1366	Ca. Caldatribacterium
Zotu1779	Acidobacteria	Zotu1468	Pelotomaculum
Zotu1978	Acidobacteria	Zotu1476	Petrimonas mucosa
Zotu2070	Syntrophorhabdus	Zotu1549	Bacteroidales group vadinHA17
Zotu2110	Acidobacteria	Zotu1555	Bacteroidales group vadinHA17
Zotu2138	Acidobacteria	Zotu1802	Petrimonas mucosa
Zotu2200	Acidobacteria	Zotu1928	Thermovirga
Zotu2302	Thermovirga	Zotu1974	Thermovirga
Zotu351	Ruminofilibacter	Zotu1978	Acidobacteria
Zotu354	Ruminofilibacter	Zotu2013	Ca. Caldatribacterium
Zotu426	Ruminofilibacter	Zotu2096	Marinimicrobia
Zotu518	Ruminofilibacter	Zotu2704	Anaerolineaceae
Zotu567	Ruminofilibacter	Zotu2919	Anaerolineaceae
Zotu607	Ruminofilibacter	Zotu3294	Cloacimonadaceae group W5
Zotu648	Ruminofilibacter	Zotu809	Treponema 2
Zotu809	Treponema 2	Zotu815	Clostridia order MBA03
Zotu933	Mesotoga infera	Zotu936	Treponema 2
Zotu950	Ruminofilibacter	Zotu958	Mesotoga infera
Zotu981	Treponema 2	Zotu981	Treponema 2

*The best possible taxonomic classification is given for each ASV.*

The cellulase activity in R1 (Table 3) showed a strong correlation to the development of ASVs representing *Clostridia* order MBA03 (3 ASVs out of the 25 best candidates), and representatives of *Lentimicrobiaceae* (4), *Ruminiclostridium* (2), *Proteiniphilum* and *Tepidanaerobacter* (2). ASVs from *Lentimicrobiaceae* (4), and *Ruminiclostridium* (1) were also identified as candidates for the xylanase activity in R1, together with other representatives of *Clostridia* (5), *Ruminicoccaceae* (4), and *Caldicoprobacter* (2). In R2 (Table 4), the cellulase activity was most similar to the relative abundances of ASVs representing *Pedosphaeraceae* (4), *Ruminococcaceae* (6), and *Ruminiclostridium* (3). Candidate ASVs relating to the xylanase activity in R2 were overall from the same taxonomic groups, with additional representation of *Spirochaetaceae* (3) and *Synergistaceae* (2). Candidate organisms relating to the cellulase activity in R3 (Table 5) were mainly uncharacterized representatives of the phylum *Acidobacteria* (8) and the genera *Ruminofilibacter* (8) and *Treponema* (3). *Treponema* (5) was also observed among the candidates for xylanase activity in R3, together with representatives of “*Candidatus* Caldatribacterium” (2), *Thermovirga* (2), and *Petrimonas mucosa* (4).

## Discussion

The present study aimed to investigate the adaptation of cellulolytic and xylanolytic microbial communities in three different digester inocula after the addition of wheat straw as a lignocellulosic substrate. The enzymatic rate and microbial community profiles were used to identify candidate organisms with potential cellulolytic and xylanolytic activity.

### Cellulolytic and Xylanolytic Adaption Depends on Inoculum Origin

Cellulose and hemicellulose are the degradable polymer groups in lignocellulose, and the rate of biogas production from lignocellulosic biomass is often considered limited by the activity of cellulases and hemicellulases (Gu et al., 2014). The consistently higher MPR in the thermophilic R1 (Figure 1) was therefore expected; partly because of the strong temperature-dependency of hydrolase kinetic rates (Ge et al., 2011), partly because R1 originates from an agricultural-based anaerobic digester, which is commonly reported as an efficient inoculum source for biogas production from lignocellulosic material (Koch et al., 2017; Liu et al., 2017). The increase in MPR (Figure 1) was accompanied by limited changes in cellulolytic (Figure 2A) and xylanolytic (Figure 2B) activities during the first 30 days of feeding (1.5 HRT), indicating that R1 initially possessed surplus hydrolytic capacity.

The significant decrease in R1 xylanase activity within the feeding period (Figure 2B) also indicates surplus xylanolytic potential, which potentially stems from a higher loading of xylan in the full-scale reactor from which the inoculum was sourced (Table 1). It is possible that other hemicellulases unable to cleave the MUF-linked substrate mimic have limited the MPR *in situ*, given the variety of hemicellulases potentially involved in lignocellulose degradation (Azman et al., 2015). Xylanase activity was selected as a representative for hemicellulose hydrolysis in the present study because xylan generally comprises a main constituent of the heterogeneous hemicellulose fraction in wheat straw (Tufail et al., 2018). The relevance of xylanolytic adaption was supported by the activity profiles in R2 and R3 (Figure 2B), which increased steadily throughout the feeding period. The slow xylanolytic adaption in R2 and R3 aligns with observations from mesophilic anaerobic digestion of rice straw (Gu et al., 2014) and biomethane potential tests of different hemicellulose polymers, including pure xylan, in manure- and sludge-based digestates (Li et al., 2018; Ma et al., 2019).

While the xylanase activity profiles developed differently in the three reactors, the cellulase activities were clearly stimulated by the addition of wheat straw in all reactors. However, the cellulase response time was significantly shorter in R3 compared to R1 and R2 (Figure 2A). The variation in response time likely reflects the differences in the microbial communities in the inocula. A tight coupling between microbial growth and cellulolytic activity has previously been reported in a pure-culture study of the *Clostridia*-affiliated *Acetivibrio cellulolyticus* (Saddler and Khan, 1980). The extended cellulolytic lag phase in R2 could hence result from a lower abundance of cellulolytic bacteria in the R2 inoculum, giving rise to pronounced growth and adaption in response to wheat straw addition. This hypothesis is supported by the strong correlations between the microbial community composition and enzymatic activities in R2. R2’s need for a longer lag phase compared to R3 was also evidenced from the MPR, which was initially lower (Figure 1). The extended lag phase in R2 was unexpected since its inoculum originated from a full-scale digester fed with various types of lignocellulosic material. A potential explanation is the higher ammonium level in R2 compared to the other reactors (Table 1), as ammonia has previously been inversely correlated with the anaerobic degradation efficiency of both straw and cellulose (Sun et al., 2016).

The stimulation of cellulase activities was only temporary as these declined in all reactors within the feeding period (Figure 2A). The enzymatic assays were conducted at conditions that approached V_*max*_ so the reported enzyme activities are measures of a reactor’s hydrolytic potential. The observed decreases in enzymatic activities consequently reflect a decrease in the concentration of active enzymes, potentially resulting from a down-regulation of the cellulase and xylanase expression. Microbial cellulase expression can both be induced and product inhibited by cellobiose and other sugar monomers (Adney et al., 1991; Kato et al., 2005) and depends on the nature of the lignocellulosic substrate (Badalato et al., 2017). Presence of acetate at concentrations of 1–2 g ⋅ L^–1^, as detected in both R2 and R3 (Table 2), has been found to reduce potential cellulolytic activity (Siegert and Banks, 2005) and growth of anaerobic cellulolytic *Bacteroidaceae* species (Saddler and Khan, 1979). However, based on the resolution of operational parameters recorded for R1, R2, and R3, we were unable to correlate the changes in enzyme potential with any of these parameters.

### Hydrolytic Adaption Results From Functional Homology in Diverse Microbes

Analyses of the microbial community compositions revealed phylogenetically diverse communities in the three reactors (Supplementary Figure 1). A previous study of microbial adaption to agricultural wastes found converging microbial community structures over time, with potential cellulose-degraders originating from the substrate (Liu et al., 2017). The phylogenetically different communities obtained in the present study stem from the inoculum-specific feed mixtures, while Liu et al. (2017) applied the same substrate mixture for all reactors. The identified potential hydrolytic bacteria suggest that the cellulolytic and xylanolytic activities are dominated by, but not constrained to, a finite number of reactor-specific phylogenetic groups.

Representatives of *Lentimicrobiaceae* and *Clostridia* order MBA03 were associated with cellulase activity in the thermophilic R1 (Table 3). These microbial groups have previously been linked to thermophilic anaerobic digestion of various substrates, including degradation of VFAs (Zheng et al., 2019), which are the primary products of lignocellulosic biomass degradation. Several of the identified *Clostridia*-affiliated ASVs were representatives of *Ruminiclostridium*, a taxonomic group that includes the known cellulose and xylan degrader, *Ruminiclostridium cellulolyticum* (Blouzard et al., 2010; Badalato et al., 2017). *Caldicoprobacter-*associated ASVs correlated to the xylanase activity in R1; this genus contains several species known to ferment xylan, including *Caldicoprobacter oshimai*, which was isolated from sheep feces (Yokoyama et al., 2010).

In the mesophilic R2, it was indicated that the ASVs affiliated with the *Pedosphaeraceae* and *Clostridia* class were important for the degradation of both cellulose and xylan (Table 4). *Clostridia* includes several known cellulolytic species (Azman et al., 2015). *Pedosphaeraceae* is not well-described in literature but has been hypothesized to participate in polysaccharide degradation during anaerobic digestion of food waste (Amha et al., 2019). Other species within the same phylum (*Verrucomicrobia*) possess a range of hydrolase-encoding genes (Martinez-Garcia et al., 2012). The R2 hydrolase correlations also identified several *Clostridia* representatives of *Ruminococcaceae*, which possesses a large genomic potential for cellulase and xylanase expression (Biddle et al., 2013).

In the sludge-based R3, several representatives of the *Acidobacterium* phylum, the genus *Ruminofilibacter* of the *Bacteriodetes* phylum, and the *Treponema* genus of the *Spirochaetes* phylum were indicated to be important for hydrolysis of the lignocellulosic wheat straw (Table 5). ASVs of the *Acidobacterium* and *Ruminofilibacter* were especially dominant in the cellulase correlations. Species within both taxonomic groups have accordingly been shown to possess cellulolytic and xylanolytic activity. Several isolated *Acidobacterium* species can use cellulose and xylan as carbon sources (Kielak et al., 2016), while *Ruminofilibacter xylanolyticum* is involved in xylan degradation (Nissilä et al., 2012). *Ruminofilibacter* species are generally poorly characterized in the literature. However, the strong correlation of several *Ruminofilibacter* ASVs with cellulase activity indicates their ability to either produce cellulases or live symbiotically with cellulase producers. Such symbiotic relationship has been proposed for the genus *Treponema*, which correlated with cellulase activity in R3. In a co-culture study, *Treponema bryantii* lived symbiotically with a cellulolytic strain of *Bacteroides succinogenes* (Stanton and Canale-Parola, 1980). The symbiotic co-culture increased cellulose degradation compared to that of the pure-culture *B. succinogenes*, despite *T. bryantii* not being able to grow on cellulose itself (Stanton and Canale-Parola, 1980). A recent study assigned *Spirochaetes* sp., including *Treponema*, to be directly involved in xylan hydrolysis (Tokuda et al., 2018). A symbiotic relationship between *Treponema* and cellulolytic *Fibrobacter* species was explained by the former exposing cellulose structures for the latter by hydrolyzing hemicellulosic structures (Xie et al., 2018). The reported roles of *Spirochaetaceae* and *Treponema* in lignocellulose degradation aligns with the strong correlation of several *Treponema* ASVs to cellulase and xylanase activity in the sludge-based R3 (Table 5); and the correlation of *Spirochaetaceae* ASVs with cellulase and xylanase activity in the manure-based R2 (Table 4). Finally, the xylanase correlations in R3 identified ASVs related to *Petrimonas mucosa*, a species that possesses hydrolytic activity against a range of carbohydrate polymers, including cellulose and xylan (Hahnke et al., 2016).

The preceding analyses established a clear link between identified ASVs and the hydrolytic activity of their phylogenetic representatives. The results consequently reveal a potential of identifying candidate organisms with cellulolytic and xylanolytic activity by linking enzymatic assays and microbial community data. This combination constitutes a promising approach to further unravel the diverse hydrolytic communities in anaerobic digestion of lignocellulosic biomass by identifying key phylogenetic groups whose functional role can be confirmed and further analyzed through targeted studies. The enzymatic-microbial approach can be combined with different metagenomic methods or advanced imaging techniques to confirm the possible link between microbial function and identity, as it has been done for other anaerobic digestion processes (Mosbæk et al., 2016; Fernando et al., 2019).

This study shows that phylogenetically different hydrolytic communities adapted from mesophilic and thermophilic inocula during anaerobic digestion of lignocellulosic wheat straw. The phylogenetic diversity was reflected in distinct activity profiles of cellulase and xylanase. Combined analyses of the microbial community and the cellulase and xylanase activity profiles were successfully used to identify potential cellulolytic and xylanolytic microorganisms. The identified microbes associated with enzymatic activity were reactor specific and included representatives of known hydrolytic groups such as *Ruminofilibacter*, *Caldicoprobacter*, *Treponema*, and a range of *Clostridia*-affiliated bacteria. The present study thus shows the potential application of enzymatic and microbial community studies to attain a deeper understanding of lignocellulosic biomass conversion in different anaerobic digestion inocula and to identify the potential hydrolytic organisms involved.

## Data Availability Statement

The sequencing datasets have been deposited in the European Nucleotide Archive (ENA) under project accession number PRJEB24105.

## Author Contributions

MJ, MD, JN, CF, LO, HM, and CK designed the study. KN and ME operated the reactors and conducted the enzymatic analyses under guidance by MJ, CF, and CK. NJ performed molecular analysis and conducted the bioinformatics analyses under guidance by JN. MJ, NJ, JN, and MK interpreted the data and wrote the manuscript with input from all co-authors. All authors contributed to the article and approved the submitted version.

## Conflict of Interest

NJ is employed by the company NIRAS A/S. The remaining authors declare that the research was conducted in the absence of any commercial or financial relationships that could be construed as a potential conflict of interest.

## Footnotes

1 https://github.com/eyashiro/AmpProc

2 https://www.rstudio.com/

## Abbreviations

ASV: Amplicon Sequence Variant
CA: Correspondence Analysis
COD: Chemical Oxygen Demand
CSTR: Continuous Stirred Tank Reactor
HRT: Hydraulic retention time
MPR: Methane Production Rate
NGS: Next-generation sequencing
OLR: Organic loading rate
RDA: Redundancy Analysis
TS: Total solids
VFA: Volatile Fatty Acid
VS: Volatile solids.
