# Supplementary material for: Cellulolytic and Xylanolytic Microbial Communities Associated With Lignocellulose-Rich Wheat Straw Degradation in Anaerobic Digestion
Source: Front Microbiol. 2021 May 25;12:645174. doi: 10.3389/fmicb.2021.645174 (PMC8186499; doi:10.3389/fmicb.2021.645174)
Supplement: Supplementary file 1 [file Data_Sheet_1.docx]

**Supplementary Information to:**

**Cellulolytic and xylanolytic microbial communities associated with lignocellulose-rich wheat straw degradation in anaerobic digestion**

Mads Borgbjerg Jensen^1✝^, Nadieh de Jonge^2,3✝^, Maja Duus Dolriis^1^, Caroline Kragelund^4^, Christian Holst Fischer^4^, Martin Rosenørn Eskesen^4^, Karoline Noer^1^, Henrik Bjarne Møller^1^, Lars Ditlev Mørck Ottosen^1^, Jeppe Lund Nielsen^2^, Michael Vedel Wegener Kofoed^1*^

**Affiliations:**

^1^ Department of Biological and Chemical Engineering, Aarhus University, Hangøvej 2, DK-8200 Aarhus N., Denmark

^2^ Department of Chemistry and Bioscience, Aalborg University, Fredrik Bajers Vej 7H, DK-9220 Aalborg E., Denmark

^3^ Niras A/S, Østre Havnegade 12, DK-9000, Aalborg C., Denmark

^4^ Danish Technological Institute, Teknologiparken, Kongsvang Allé 29, DK-8000 Aarhus C., Denmark

^✝^The authors contributed equally to the study and share first authorship.

^*^Correspondence:

Michael Vedel Wegener Kofoed

[mvk@bce.au.dk](mailto:mvk@bce.au.dk)

**
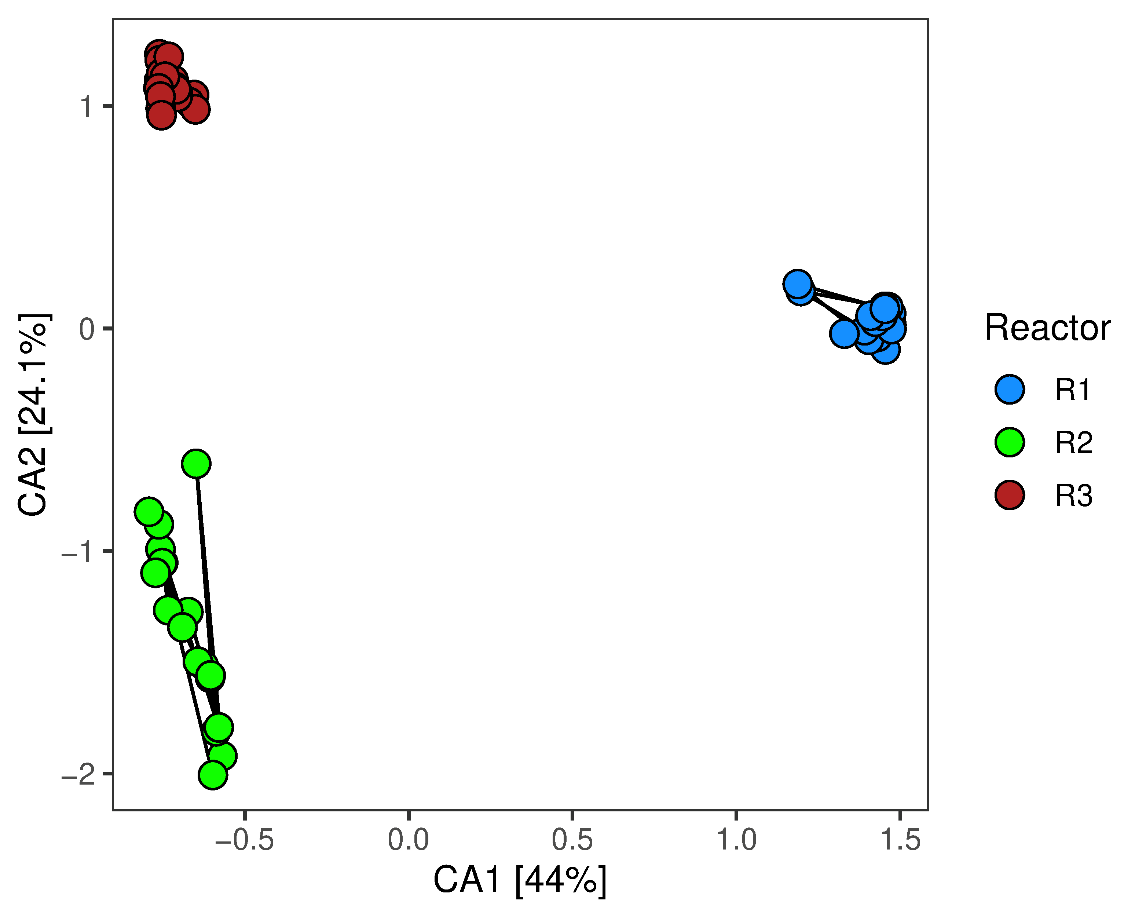
**

**Figure S1:** Beta diversity. Correspondence analysis of the three duplicate reactors. Samples are coloured by reactor, and a line is drawn between consecutive points in the time series. R1 is thermophilic, and R2 and R3 are mesophilic reactors.

**
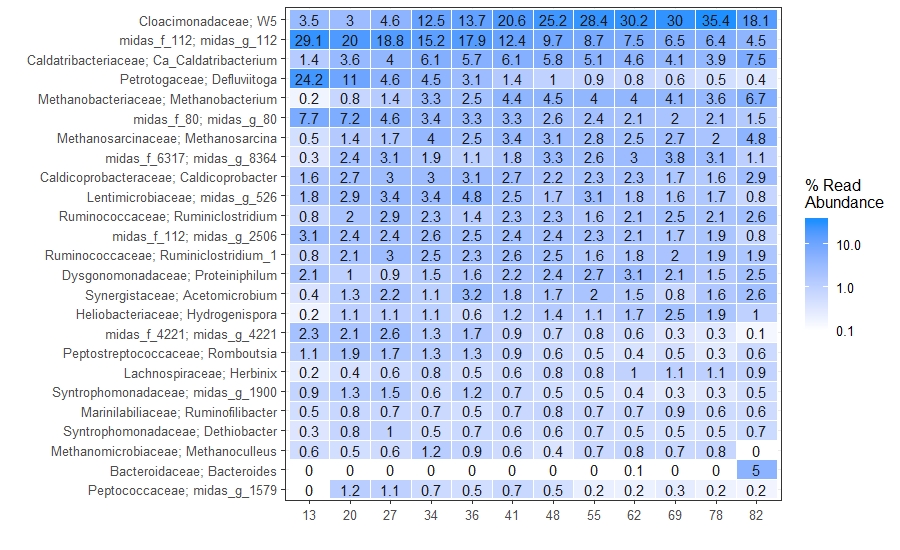
**

**Figure S2:** The 25 most abundantly observed taxonomic groups in the thermophilic R1, at genus level. Data is shown as the average relative abundance between the duplicate reactors.

**
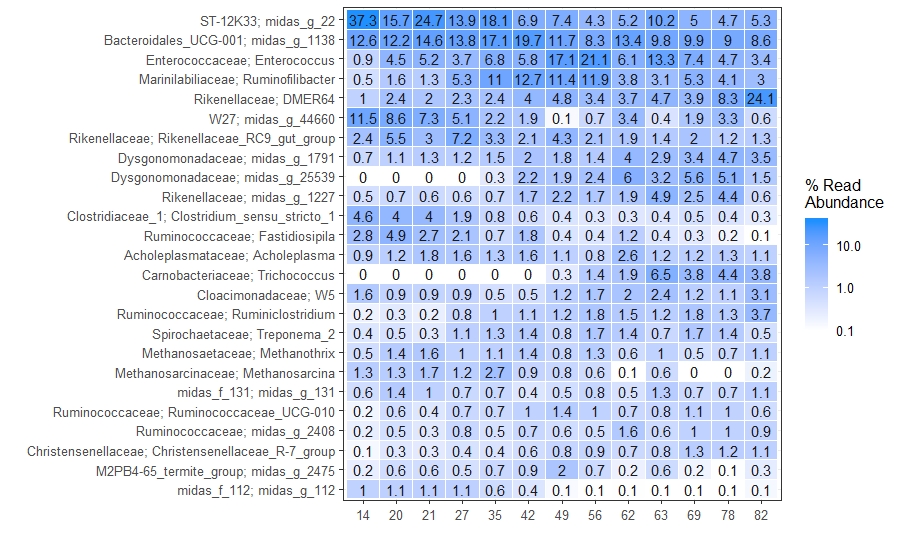
**

**Figure S3:** The 25 most abundantly observed taxonomic groups in the mesophilic R2, at genus level. Data is shown as the average relative abundance between the duplicate reactors.

**
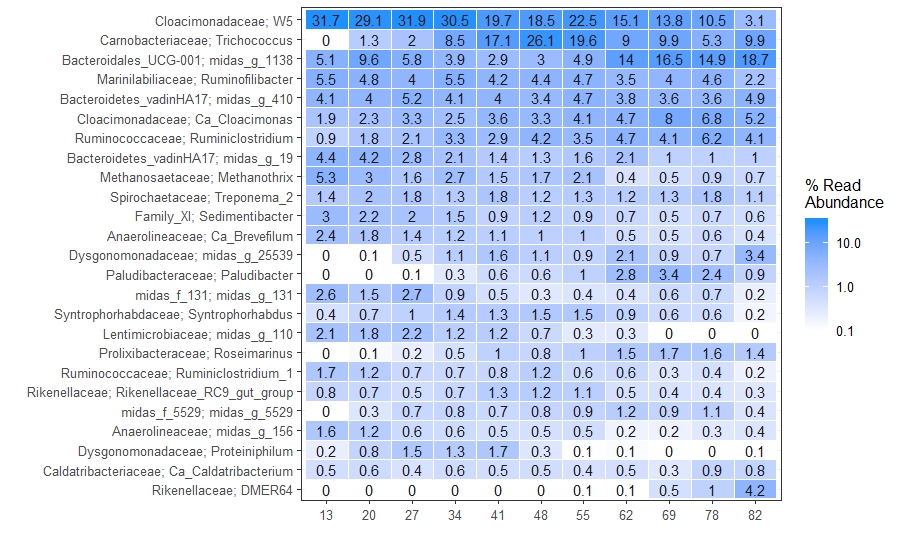
**

**Figure S4:** The 25 most abundantly observed taxonomic groups in the mesophilic R3, at genus level. Data is shown as the average relative abundance between the duplicate reactors.

**
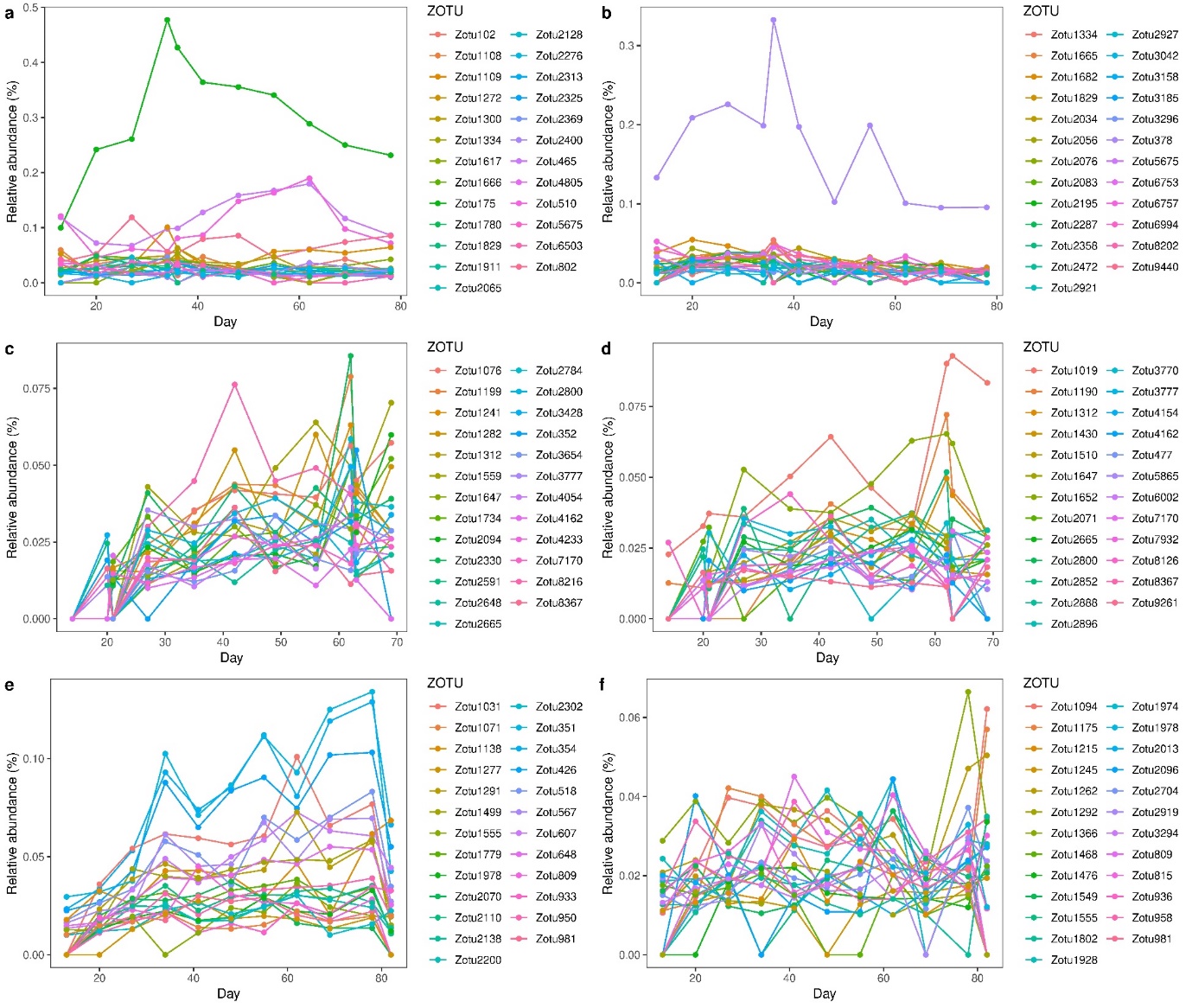
**

**Figure S5:** The 25 ASVs in the duplicate reactors R1 (a,b), R2 (c,d), and R3 (e,f), with the strongest relationship to the cellulase (a,c,e) or xylanase (b,d,f) activity. R1 is thermophilic, and R2 and R3 are mesophilic reactors.
